# Supplementary material for: Nodes Having a Major Influence to Break Cooperation Define a Novel Centrality Measure: Game Centrality
Source: PLoS One. 2013 Jun 28;8(6):e67159. doi: 10.1371/journal.pone.0067159 (PMC3696096; doi:10.1371/journal.pone.0067159)
Supplement: Text S1 — This supporting information (Text S1) contains a supplementary figure, 2 supplementary tables, a detailed description of the NetworGame algorithm for the simulation of spatial social dilemma games with pseudocodes, as well as 11 supplementary references. (PDF) [file pone.0067159.s001.pdf]

Supporting Information (Text S1)

Nodes having a major influence to break cooperation define a novel centrality measure: game centrality

Gabor I. Simko[1,2], Peter Csermely[1,*]

1 Department of Medical Chemistry, Semmelweis University, Budapest, Hungary,
2 Vanderbilt University, Nashville, TN USA

*Corresponding author, E-mail: csermely.peter@med.semmelweis-univ.hu

Summary

In this Supporting Information (Text S1) we give a detailed description of the NetworGame spatial social dilemma game simulation program package. Besides the pseudocode description of the NetworGame algorithm the Supporting Information also contains a supplementary figure, 2 supplementary tables as well as 11 references.

The computer programs of the NetworGame package with a User Guide can be downloaded from here: www.linkgroup.hu/NetworGame.php.

Table of Contents

Supplementary Figure S1.. 2
Supplementary Tables.. 3
Table S1. List of consensus party hubs.. 3
Table S2. List of consensus date hubs.. 4
Description of the NetworGame algorithm.. 5
Supplementary References.. 8

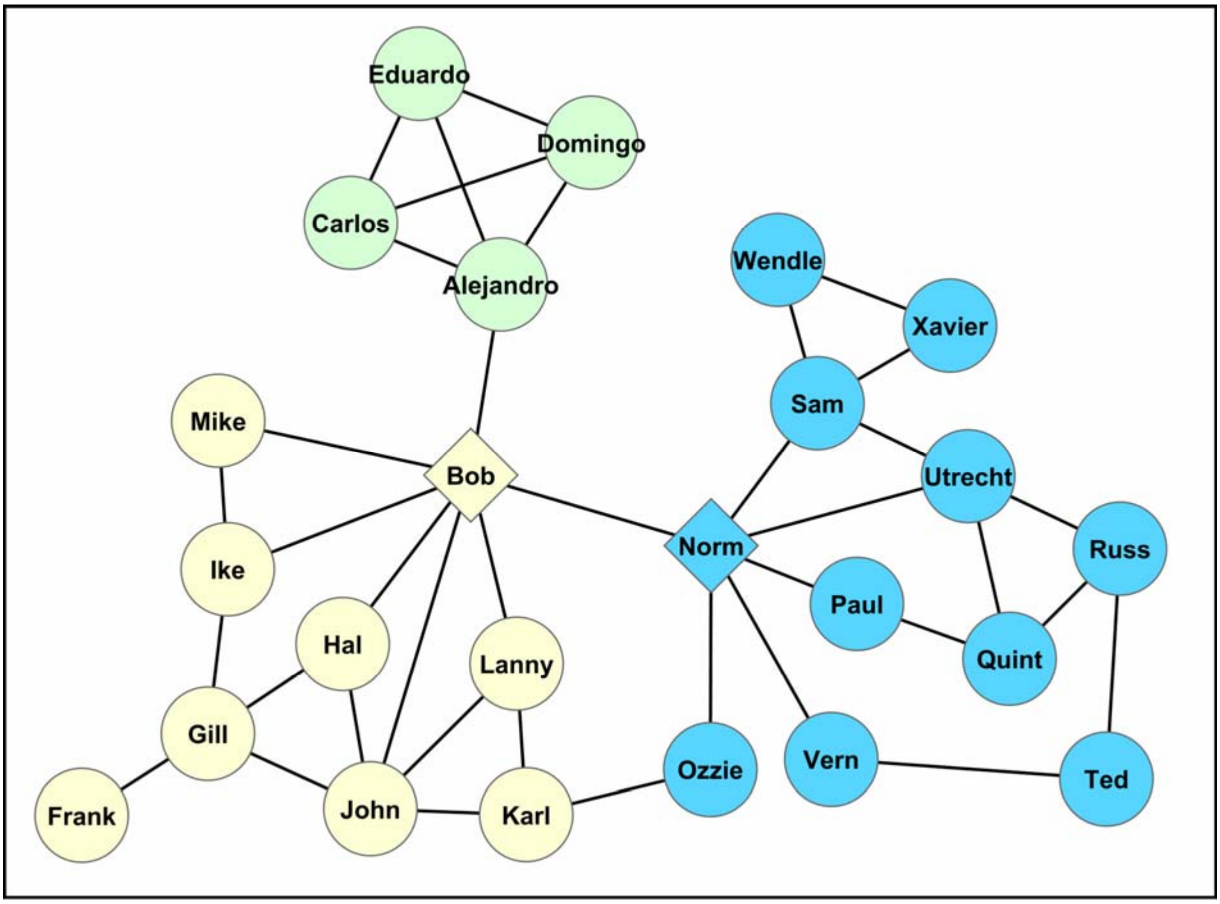

Supplementary Figure S1. Michael's strike network [1]. The three worker groups of a former forest product manufacturing factory containing younger, English-speaking (yellow); older, English-speaking (blue); or younger, Spanish-speaking workers (green) were marked. Sam and Wendle (top right) were the union leaders, who failed to break the strike, while Bob and Norm (center, marked with diamonds) were the pair of workers, who successfully broke the strike.

Supplementary Tables

Table S1. List of consensus party hubs

**Consensus party hub ORFs**[a]	YHR077C
YAR002W	YHR089C
YAR003W	YHR166C
YBL004W	YHR200W
YBL007C	YIL115C
YBL038W	YJR045C
YBL050W	YJR065C
YBL084C	YJR068W
YBL099W	YJR121W
YBR010W	YKL018W
YBR084W	YKL022C
YBR087W	YKL068W
YBR118W	YKL085W
YBR245C	YKL129C
YDL029W	YLR127C
YDL065C	YLR212C
YDL134C	YMR080C
YDL208W	YMR109W
YDL213C	YMR116C
YDR103W	YNL016W
YDR118W	YNL094W
YDR244W	YNL102W
YDR264C	YNL138W
YDR395W	YNL172W
YER157W	YNL290W
YFR002W	YOL094C
YFR036W	YOR157C
YGL004C	YOR249C
YGL153W	YOR250C
YGL200C	YOR270C
YHL030W	YPL213W
YHR016C	YPR088C

[a]The open reading frame names of 63 consensus yeast party hubs were determined and listed as in [2] comparing the party hubs of the high fidelity yeast protein-protein interaction network [3] with those published in other 5 publications [4-8], and listing only those as ‘consensus party hubs’, which were never classified as a date hub.

Table S2. **List of consensus date hubs**

**Consensus date hub ORFs**[a]	YER095W	YKL081W	YNL093W
YAL005C	YER110C	YKL095W	YNL127W
YBL016W	YER148W	YKL104C	YNL135C
YBL023C	YER155C	YKL166C	YNL243W
YBL093C	YER165W	YKL203C	YNL263C
YBL105C	YFL017W-A	YKR001C	YNL271C
YBL106C	YFR021W	YKR026C	YNL298W
YBR011C	YFR028C	YKR068C	YOL086C
YBR089C-A	YFR034C	YLL021W	YOL090W
YBR114W	YGL003C	YLL026W	YOL108C
YBR119W	YGL092W	YLL039C	YOL123W
YBR126C	YGL116W	YLR044C	YOL133W
YBR135W	YGL198W	YLR096W	YOL135C
YBR160W	YGL207W	YLR180W	YOR039W
YBR175W	YGR009C	YLR229C	YOR089C
YBR254C	YGR040W	YLR310C	YOR106W
YBR274W	YGR086C	YLR319C	YOR212W
YBR279W	YGR104C	YLR337C	YOR244W
YCR009C	YGR134W	YLR342W	YOR304W
YDL047W	YGR218W	YLR423C	YOR308C
YDL101C	YGR274C	YLR452C	YPL004C
YDL126C	YHR061C	YML007W	YPL031C
YDL160C	YHR099W	YML010W	YPL082C
YDL188C	YHR152W	YML064C	YPL129W
YDR142C	YIL038C	YML109W	YPL153C
YDR155C	YIL046W	YMR001C	YPL161C
YDR170C	YIL094C	YMR012W	YPL181W
YDR172W	YJL081C	YMR043W	YPL248C
YDR192C	YJL095W	YMR054W	YPL256C
YDR216W	YJL138C	YMR125W	YPR054W
YDR238C	YJL141C	YMR139W	YPR072W
YDR240C	YJL164C	YMR199W	YPR086W
YDR309C	YJL187C	YMR201C	YPR107C
YDR473C	YJL194W	YMR213W	YPR119W
YDR523C	YJR066W	YMR273C	YPR182W
YEL009C	YJR090C	YMR304W	
YER081W	YJR091C	YNL006W	

[a]The open reading frame names of 145 consensus yeast date hubs were determined and listed as in [2] comparing the date hubs of the high fidelity yeast protein-protein interaction network [3] with those published in other 5 publications [4-8], and listing only those as ‘consensus date hubs’, which were never classified as a party hub.

Description of the NetworGame algorithm

The 2.0 version of the NetworGame program is an updated version of the NetworGame 1.0 version published in a preliminary conference report [9]. NetworGame 2.0 is available in our web-site (www.linkgroup.hu/NetworGame.php). The 2.0 version utilizes our experiences gained with the 1.0 version. The NetworGame 2.0 program package is a cross-platform, generic tool to simulate repeated spatial games. This simulation program includes i.) options for pay-off matrices of any symmetric normal form games (with 2 strategies); ii.) several well-known, replicator-type strategy update rules, as well as the option for additional, user-defined strategy update rules in a 'plugin'-type format; iii.) synchronous, and semi-synchronous updating [10]; iv.) and the option for the inclusion of any real world networks in a Pajek format [11].

Here we provide the pseudocode for the algorithm, which describes the flow of the program and the effects of the configuration parameters. A User Guide of version 2.0 can be downloaded from here: www.linkgroup.hu/NetworGame.php.

Configurator

- testNode and testEdge are configuration parameters
- printSteps, printStepsStdDev and printLast are configuration parameters
- Nodes and Edges represent the network, where Edges is a set of pairs (src,dst)

```
initialize payoff matrix
if (testNode specified) then
  for i in Nodes do
    initialize strategies
    Si = testNode
    run simulations
    print statistics
  end
else if (testEdge specified) then
  for (src,dst) in Edges do
    initialize strategies
    Ssrc = testEdge
    Sdst = testEdge
    run simulations
    print statistics
  end
else
  initialize strategies
  run simulations
  if (printSteps) then print step-wise average cooperation levels
  if (printStepsStdDev) then print step-wise standard deviances
  if (printLast) then print average cooperation for each node at last step
end
```

Run simulations

- M is a set of simulations
- L is number of steps
- n is the size of M
- memUsage and elapsedTime are internal variables representing the current memory usage of the system and the elapsed time since the start of the simulations
- maxError is a parameter controlling the statistical accuracy
- numberofsimulations, numberofsteps, mem and time are configuration parameters

```
if (numberofsimulations specified) then
  n = numberofsimulations, i.e. M has size of numberofsimulations
else
  n = 100, i.e. M has size of 100 initially, but it can grow
end

if (numberofsteps specified) then
  L = numberofsteps is the number of steps in each simulation
else
  L = 101 (and it can grow)
end
```

- *run simulations until we reach the specified resource limits, or get below the desired statistical error*

```
while (memUsage < mem and elapsedTime < time) do
  for m in M do
    simulate m up to steps L
  end
  A_i = average cooperation at step i for each m in M
  currMean = average of A_x, where x=L-50 to L
  prevMean = average of A_x, where x=L-150 to L-100
  stddev   = standard deviation of A_x, where x=L-50 to L
  meanerror = sqrt(stddev / n*(n-1))
  if (numberofsteps unspecified and abs(prevmean-currmean) > maxError) then
    L = L + 1
  else if (numberofsimulation unspecified and meanerror > maxError/2) then
    add a simulation to M (inherently n = n + 1)
  else
    finish simulations
  end
end
```

- *calculate statistics for printing*

```
for i = 1,2...,L do
  calculate average cooperation at step i over all m in M
  calculate standard deviation at step i over all m in M
end

for n in Nodes do
  calculate average cooperation at last step for n over all m in M
end
```

Simulate m up to steps L

- S_i is the current strategy of node i
- P_i is the current payoff of node i
- useWeights, x0 and x1 are weight parameters controlling the effect of edge weights
- Neighbors(i) is the set of neighbors for node i
- Payoff[i,j] is the payoff matrix value when strategy j plays against strategy j
- payoffSchema is a configuration parameter

```
for n = 1,2,...,L do
  - simulating current round and calculating payoffs
    for i in Nodes do
      P_i = 0
      counter = 0
  - the probability of a game is dependent on the weight parameters and edge weight W_i,j
      for j in Neighbors(i) do
        if (not useWeights or random(0,1) <= (W_i,j-x0)/(x1-x0)) then
          P_i = P_i + Payoff[S_i,S_j]
          counter = counter + 1
      end
      if (payoffSchema = degree or payoffSchema = averaging) then
        P_i = P_i / counter (if counter > 0)
    end
  - updating strategies (strategyUpdateRule can be implemented as a plugin, it may have memory,
    or might be one of the built-in rules: best takes over or proportional update
    for k in Nodes do
      S_k = strategyUpdateRule(...)
    end
end
```

Supplementary References

1. Michael JH (1997) Labor dispute reconciliation in a forest products manufacturing facility. Forest Products J. 47: 41–45.
2. Kovács IA, Palotai R, Szalay MS, Csermely P (2010) Community landscapes: a novel, integrative approach for the determination of overlapping network modules. PLoS ONE 7: e12528.
3. Ekman D, Light S, Björklund A, Elofsson A (2006) What properties characterize the hub proteins of the protein-protein interaction network of *Saccharomyces cerevisiae*? Genome Biol. 7: R45.
4. Han JD, Bertin N, Hao T, Goldberg DS, Berriz GF, et al. (2004) Evidence for dynamically organized modularity in the yeast protein-protein interaction network. Nature 430: 88–93.
5. Batada NN, Reguly T, Breitkreutz A, Boucher L, Breitkreutz BJ, et al. (2006) Stratus not altocumulus: a new view of the yeast protein interaction network. PLoS Biol. 4: e317.
6. Kim PM, Lu LJ, Xia Y, Gerstein MB (2006) Relating three-dimensional structures to protein networks provides evolutionary insights. Science 314: 1938–1941.
7. Komurov K, White M (2007) Revealing static and dynamic modular architecture of the eukaryotic protein interaction network. Mol. Syst. Biol. 3: 110.
8. Bertin N, Simonis N, Dupuy D, Cusick ME, Han JD, et al. (2007) Confirmation of organized modularity in the yeast interactome. PLoS Biol. 5: e153.
9. Farkas IJ, Korcsmáros T, Kovács IA, Mihalik Á, Palotai R, et al. (2011) Network-based tools in the identification of novel drug-targets. Science Signaling 4: pt3.
10. Saif MA, Gade PM (2009) The prisoner’s dilemma with semi-synchronous updates: evidence for a first-order phase transition. J. Stat. Mech. Theor. Exp. 7: P07023.
11. Batagelj V, Mrvar A (1998) Pajek - Program for large network analysis. Connections 21: 47–57.
